# Autistic Symptoms in Children and Adolescents with Gender Dysphoria

**DOI:** 10.1007/s10803-017-3417-5

**Published:** 2017-11-30

**Authors:** Anna I. R. van der Miesen, Annelou L. C. de Vries, Thomas D. Steensma, Catharina A. Hartman

**Affiliations:** 1 Center of Expertise on Gender Dysphoria, VU University Medical Center, PO box 7057, 1007 MB Amsterdam, The Netherlands; 2 Department of Medical Psychology, VU University Medical Center, Amsterdam, The Netherlands; 3 Department of Psychiatry, University of Groningen, University Medical Center Groningen, Groningen, The Netherlands

**Keywords:** Autism spectrum disorder, Comorbidity, Co-occurrence, Gender dysphoria, Gender identity disorder

## Abstract

Studies have shown an increase of symptoms of autism spectrum disorder (ASD) in gender dysphoria (GD). Various hypotheses try to explain this possible co-occurrence (e.g., a role of resistance to change, stereotyped behaviors or prenatal testosterone exposure). This study examined ASD symptoms with the Children’s Social Behavior Questionnaire (CSBQ) in 490 children with GD compared to 2507 typically developing (TD) and 196 children with ASD. CSBQ total scores of the GD sample were in between scores from the TD and ASD sample. The GD sample showed elevated levels of autistic symptomatology on all subdomains, not only on stereotyped and resistance to change. Further, no gender differences and interaction effects were found on the total CSBQ, making a sole role for prenatal testosterone unlikely.

## Introduction

Symptoms of gender dysphoria (GD) as defined in the DSM-5 are a marked incongruence between one’s experienced and assigned gender along with a persistent and strong desire to be of the other gender (APA 2013). The estimated prevalence rates of GD in adults are 1:10,000–1:20,000 for males and 1:30,000–1:50,000 for females (Zucker and Lawrence 2009) and a recent meta-analysis of Arcelus et al. (2015) reported a prevalence of GD in adults of 4.6:100,000. Autism spectrum disorder (ASD) consists of problems in social communication and interaction in addition to repetitive behavior and specific interests (APA 2013). In children, the overall prevalence of ASD is estimated at 1%; 1:42 for boys and 1:189 for girls respectively (Lai et al. 2014). The current paper will refer to boys/men or girls/women when assigned gender at birth is male respectively female, which may be incongruent from the experienced gender in the case of GD.

There are several case reports on individuals with both ASD and GD (e.g., Landen and Rasmussen 1997; Lemaire et al. 2014). The first systematic study that focused on the incidence of an ASD diagnosis in children and adolescents referred to a specialized gender identity clinic (de Vries et al. 2010) reported an ASD rate, by using a diagnostic interview, of 7.8%. This is higher than expected based on the prevalence in the general population (Fombonne 2005; de Vries et al. 2010), although comparison with a clinical control group was lacking. The study additionally demonstrated an overrepresentation of ASD diagnoses in boys compared to girls with a ratio of 3:1 (de Vries et al. 2010).

Three subsequent studies on GD and ASD focused on symptoms of ASD (instead of an ASD diagnosis) in GD referred samples (Jones et al. 2012; Pasterski et al. 2014; Skagerberg et al. 2015). Pasterski et al. (2014) investigated adults with GD using the Autism Spectrum Quotient (AQ; Woodbury-Smith et al. 2005). Utilizing the threshold for a potential diagnosis, an ASD-rate of 5.5% was found with no significant gender difference in mean AQ. Jones et al. (2012) compared adults with GD, typical adults and adults diagnosed with ASD. While also using the AQ to measure autistic traits, in this study, the Broader Autistic Phenotype (BAP) was investigated. The BAP is defined more broadly than the more circumscribed ASD phenotype as a subclinical set of traits or characteristics that index familiality to ASD (Woodbury-Smith et al. 2005). 17.5% of the GD sample had a score above the AQ cut-off for BAP, with, in contrast to the male–female distribution in ASD, more females with GD scoring above the cut-off than males with GD. Skagerberg et al. (2015) measured autistic symptoms in children and adolescents with GD using the Social Responsiveness Scale (SRS; Constantino and Gruber 2005). The SRS is a quantitative measure of autistic symptoms and the total score is divided into three subgroups (severe, mild/moderate and normal range). Skagerberg et al. found ASD scores that fell, on average, in the mild/moderate range in the GD sample and in the normal range in a typical developing (TD) sample. No significant difference between boys and girls with GD was found. Only one study examined risk factors for ASD in children with GD. This study used the SRS to measure ASD symptoms and found that 44.9% scored in the severe or mild/moderate range (VanderLaan et al. 2015a). VanderLaan et al. used the Gender Identity Questionnaire for Children to measure gender nonconformity (Johnson et al. 2004) and coded for putative risk factors for ASD of advanced parental age, high male:female-sibling sex ratio and high birth weight. It was found that only high birth weight, but not the other risk factors, was associated with both high gender nonconformity and autistic traits among children with GD.

While these aforementioned studies examined ASD symptoms in GD samples, only two studies of children (Janssen et al. 2016; Strang et al. 2014), and one study of adults (Dewinter et al. 2017) took the converse approach and investigated GD symptoms within an ASD population. Children with ASD were compared to non-referred controls and were asked for feelings of gender variance by one item of the Child Behavior Checklist (CBCL): “Wishes to be of the other gender” (Achenbach and Rescorla 2001). Strang et al. (2014) found that children with ASD were 7.59 times more likely to express gender variance compared to their typically developing peers, and the gender variance occurred equally in boys and girls. Janssen et al. (2016) found that children with ASD were 7.76 times more likely to express gender variance than children from the non-referred comparison group, with no significant difference between boys and girls. In the study in adults with ASD, Dewinter et al. found that in both men and women 0.9% identified as those opposite of their assigned gender at birth. About 22% of women and 8% of the men with ASD reported some gender non-conforming feelings. As no reference data were available, no statistical comparisons with estimates from the general population could be made (Dewinter et al. 2017).

Thus, although specific findings and methodology differ somewhat from one study to the other, almost all found increased symptoms of ASD in individuals with GD and vice versa. Several hypotheses have been put forward to understand these findings (for an overview see van der Miesen et al. 2016). In the theory of the extreme male brain (EMB), it is posited that individuals with ASD demonstrate an extreme of the typical male pattern of behaviors and cognitions originating from high levels of fetal testosterone (Baron-Cohen 2002). Fetal levels of testosterone are also suggested to be related to (symptoms of) GD, especially in assigned girls at birth, explaining their male identity and behavior. Findings of the study of Jones et al. (2012) supported the prediction of the EMB that females with GD would show more autistic traits than males with GD. Apart from the EMB theory, it has been hypothesized that one specific subdomain of the cluster B repetitive or obsessive symptoms of the autistic spectrum, i.e., rigidity or resistance to change, might specifically contribute to this possible co-occurrence (APA 2013; de Vries et al. 2010). In typical gender identity development, young children demonstrate more rigidity than older children with respect to gender identity but after age 5 years this rigidity generally decreases (Ruble et al. 2007). It is hypothesized that individuals with ASD might not reach a certain level of flexibility in gender development necessary to deal with gender variant feelings, which might lead to the overrepresentation of ASD in GD (de Vries et al. 2010). Others have suggested a link between obsessions in GD and ASD (Gallucci et al. 2005; Parkinson 2014; VanderLaan et al. 2015b; Williams et al. 1996). For example, in a case report about an assigned male at birth, it was suggested that stereotyped cross-gender behavior (pre-occupation with feminine cross-dressing and bright and shiny objects) might be attributed to these co-occurrence (Williams et al. 1996). VanderLaan et al. (2015b) investigated these intense interests and obsessions in children with GD. Compared to their non GD siblings, boys with GD showed more obsessional interests in both gender-related and non-gender related subjects. It was suggested that the clinical presentation of GD might arise because of the contribution of obsessional cross-gender interests stemming from ASD. Parkinson (2014) described two young men with ASD and the wish for medical gender reassignment treatment. In both cases, feelings of GD desisted and it was cautioned that apparent GD might actually be a transient obsessive preoccupation related to ASD. Another possibility might be that social impairments in individuals with GD are not actual ASD symptoms but stem from stress due to sexual minority status and stigma (Baams et al. 2013; Holt et al. 2014; Skagerberg et al. 2015).

In summary, studies found an overrepresentation of (symptoms of) ASD in individuals with GD and vice versa but so far have focused either on symptom levels of ASD in adults with GD (Jones et al. 2012; Pasterski et al. 2014), or autistic symptoms in children and adolescents without the use of a control sample with ASD (Skagerberg et al. 2015) or focused only on ASD diagnoses (de Vries et al. 2010). A focus on diagnosis is less sensitive to the presence of subthreshold or mild autistic symptoms, which may be highly relevant in this group. As with regard to underlying mechanisms, two hypotheses have been posited. Some prior results supported the theory of the EMB (Jones et al. 2012), although other findings did not (de Vries et al. 2010; Pasterski et al. 2014; Skagerberg et al. 2015; Strang et al. 2014). Others have suggested overlap particularly on the subdomain of rigidity or repetitive and obsessive behaviors of the autistic spectrum (APA 2013; de Vries et al. 2010; Gallucci et al. 2005; Parkinson 2014; VanderLaan et al. 2015b; Williams et al. 1996) or only in the social domain (Holt et al. 2014).

Therefore, in a large sample of children and adolescents with GD, we examined the presence of autistic symptoms in children and adolescents with GD and compared these to symptom levels in typically developing (TD) children and adolescents and children and adolescents with ASD. We expected to find, first, higher levels of autistic symptoms in children and adolescents with GD than in TD developing children and adolescents. Second, we predicted that symptoms of specific subdomains (stereotyped behavior or obsessions) of the autistic spectrum would be particularly elevated in this sample. Third, we tested whether gender differences that could support the EMB theory exist in our sample by investigating gender differences and the interactions by gender between the groups with respect to ASD symptoms in children and adolescents with GD compared to their TD peers.

## Methods

### Participants and Procedure

The current study included a total of 3245 participants. A sample of children and adolescents with GD (N = 542) were investigated and compared to two groups from a Dutch normative study: children and adolescents with ASD (N = 196) and TD children and adolescents (N = 2507) (Hartman et al. 2006, 2015).

Between March 2005 and December 2012, the 542 children and adolescents with GD were consecutively referred to the Center of Expertise on Gender Dysphoria of the VU University Medical Center in Amsterdam and included in the study. Children and parents had various sessions with a trained psychologists and/or psychiatrists and filled out questionnaires (de Vries and Cohen-Kettenis 2012). Mean IQ in the GD sample was 99.53 (SD = 14.61; Wechsler 1997; Wechsler et al. 2002). At the time of the study, the DSM-5 criteria for GD were not published yet. Diagnosis was made based on the DSM-IV-TR criteria for Gender Identity Disorder (GID; APA 2000). As GD is currently considered to be the preferred terminology, we use the term GD instead of GID. Fifty-two participants were unable to complete the diagnostic protocol, and did therefore not participate in this study. Reasons for drop out were severe interfering psychosocial problems or desistance of GD symptoms. This resulted in a total group of 490 participants with GD (mean age = 11.1, SD = 3.73), 248 boys (mean age = 10.1, SD = 3.79) and 242 girls (mean age = 12.1, SD = 3.39). Included participants did not differ from not included participants with regard to age (χ^2^ = 2.698, *p* = 0.259) and IQ (*F* = 0.23, *p* = 0.631). However, significantly more boys were not included than girls (χ^2^ = 12.89, *p* = 0.001). The ethical committee approved this study and all parents gave written informed consent as well as all adolescents above age 11 years.

The first comparison group consisted of 2507 TD children and adolescents (mean age = 10.1, SD = 3.73), 1248 boys (mean age = 10.2, SD = 3.72) and 1259 girls (mean age = 10.1, SD = 3.73). This normative sample was recruited between June 1996 and December 2000 from primary and secondary schools in the Netherlands (Hartman et al. 2015). Caregivers of these children were approached through randomly selected schools.

The second comparison group consisted of 196 children and adolescents (mean age = 10.8, SD = 3.08), 100 boys (mean age = 10.8, SD = 2.96) and 96 girls (mean age = 10.7, SD = 3.23) with an ASD diagnosis. This group was clinically referred for diverse behavioural, emotional and developmental problems between June 1996 and December 2000 to a child and adolescent psychiatry clinic in the Netherlands (Hartman et al. 2006). Child and adolescent psychiatrists made the DSM-IV classifications after their diagnostic procedures (Hartman et al. 2006). These procedures included clinical interviews, in which caregivers described the present functioning of their children and the developmental history. Play sessions with children provided additional information and staff officials provided information about the children’s behavior at school.

### Measures

#### Autistic Symptoms

The Children’s Social Behaviour Questionnaire (CSBQ) was used to investigate symptoms of ASD and was completed by parents or caregivers (Hartman et al. 2006, 2015). The CSBQ consists of 49 items on a 3-point Likert scale regarding different symptoms of ASD and has six subscales:

1. The *tuned* subscale assesses the extent of situation appropriate behavior and emotions. This subscale contains of 11 items and an example is “Does not know when to stop”. Higher scores represent child characteristics such as being overly stubborn or persistent angriness.
2. The *social* subscale measures responses to social contact, social needs and initiation of contact. This subscale includes 12 items, for example: “Lives in a world of his/her own”. Children who have a high score on this subscale show less reciprocal behavior and less social interest.
3. The *orientation* subscale assesses orientation in activity, time and place. This subscale consists of eight items. Examples of items are: “Does things without realizing the aim” and “Gets lost easily”. Children who have a high score on this subscale lack the overview of activities and situations.
4. The *understanding* subscale measures the ability to understand social information with respect to the use of language and communication. This subscale contains seven items. An example of an item is: “Does not understand jokes”.
5. The *stereotyped* subscale assesses the occurrence of stereotyped movements and atypical responses to information from the senses. This subscale includes eight items, for instance: “Smells objects” and “Sways to and fro”. Children who have a high score on this subscale are for example very sensitive to certain sounds or other input to the senses and make unusual movements with their hands and body.
6. The c*hange* subscale represents aspects related to the feeling of fear and resistance to change. This subscale consists of three items and an example is: “Opposes changes”. Children with a high score on this subscale react strongly to new situations and stick to routines.

The reliability and validity of the CSBQ were considered good and included estimates for test–retest, internal, and inter-rater reliability, and for validity with criterion measures (e.g. theory of mind, diagnostic outcome) (de Bildt et al. 2009; Hartman et al. 2006, 2015). Although the CSBQ is not a diagnostic instrument, a threshold total CSBQ score of 38 or higher was indicated as suggestive for a possible DSM classification of ASD which corresponds to the 96.5th percentile in the current TD sample (Luteijn et al. 2002).

### Analyses

First, between group differences were analyzed using multivariate general linear modelling (GLM) on the six scales of the CSBQ simultaneously. Second, a multivariate GLM analysis with assigned gender at birth and a gender by group interaction as additional predictors was used to identify possible gender differences. These multivariate tests were followed up by univariate GLM analyses per subscale of the CSBQ. Cohen’s *d* was used to measure effect sizes between the GD group and comparison groups (Cohen 1988). An effect size of 0.80 or larger was considered as large, 0.50–0.79 as medium, and 0.20–0.49 as small, and an effect size smaller than 0.20 as negligible. For subscales that differed among the groups, post-hoc *t* tests were applied to further characterize the scores of the GD group relative to normative TD behavior as well as to the scores typical for children diagnosed with ASD.

## Results

### Children and Adolescents with Gender Dysphoria Versus Comparison Groups

Table 1 shows the mean total CSBQ (sub-)scores per sample. On average, the scores of the children and adolescents with GD were all in between the scores of TD children and adolescents and those diagnosed with ASD. A multivariate GLM analysis with group as a fixed factor and the CSBQ subscales as the dependent measures showed an overall difference using Pillai’s Trace (*F* = 689.68; *df* = 7; *p* < 0.001). Subsequent univariate GLM analyses for the total CSBQ, and the CSBQ subscales separately, indicated that groups differed from each other on the total CSBQ scale (*F* = 587.15; *df* = 2; *p* < 0.001) as wel as on all subscales (all six univariate *p* values < 0.001). Post-hoc analysis showed that children and adolescents with GD had significantly higher mean scores on all subscales as well as on the total CSBQ in comparison with the TD sample. In addition, children and adolescents with GD had significantly lower scores than those diagnosed with ASD on the total CSBQ score and on all subscales. In terms of effect sizes, these differences tended to be large when comparing scores of GD with those of children and adolescents diagnosed with ASD (mean Cohen’s *d* across subscales: 1.00; see further Table 1 for effect sizes per subscale) and medium when scores of children and adolescents with GD were compared to those of TD children and adolescents (mean Cohen’s *d* across subscales: 0.52).

**Table 1:** Mean CSBQ scores for children and adolescents with gender dysphoria and comparison groups

	Typically developing		Gender dysphoric		ASD		Statistical analysis^a^		Effect sizes Cohen’s *d* ^c^	
Scales^d^	Mean	SD	Mean	SD	Mean	SD	F^b^	*p* values	GD vs. TD	GD vs. ASD
Tuned	4.20	4.11	7.31	5.50	11.98	5.90	341.855	< .001	0.71	0.83
Social	1.75	2.66	4.03	4.49	8.48	5.08	466.620	< .001	0.75	0.95
Orientation	1.86	2.46	2.97	3.27	7.19	3.92	364.628	< .001	0.42	1.21
Understanding	2.04	2.34	3.29	3.13	7.55	3.83	434.310	< .001	0.50	1.27
Stereotyped	1.08	1.85	1.64	2.45	3.62	3.63	138.780	< .001	0.29	0.70
Change	0.76	1.21	1.33	1.73	3.25	2.00	315.983	< .001	0.43	1.06
CBSQ total	11.69	11.49	20.58	15.71	42.08	16.72	587.150	< .001	0.72	1.34

*GD* children and adolescents with gender dysphoria, TD typically developing children and adolescents, *ASD* children and adolescents with ASD, *CSBQ* Children’s Social Behaviour Questionnaire

^a^Additional post-hoc analyses comparing the sample with gender dysphoria with the typically developing and ASD sample demostrated that on all subscales as well as on the total score, children and adolescents with gender dysphoria had a significantly higher score than typically developing children and a significantly lower score than children with ASD

^b^ *df* = 2

^c^Effect sizes Cohen’s *d*: 0.80 or higher is a large effect size, 0.50–0.79 a medium effect size and 0.20–0.49 small. Effect sizes less than 0.20 are negligible (Cohen 1988)

^d^ *Tuned* behavior not optimally changed to the situation, *Social* reduced social interest and contact, *Orientation* orientation problems, in activity, place of time, *Understanding* difficulties in understanding social information, *Stereotyped* stereotyped behavior, *Change* fear or resistance to changes

### Gender Differences Within Children and Adolescents with Gender Dysphoria Versus Comparison Groups

**Fig. 1:**
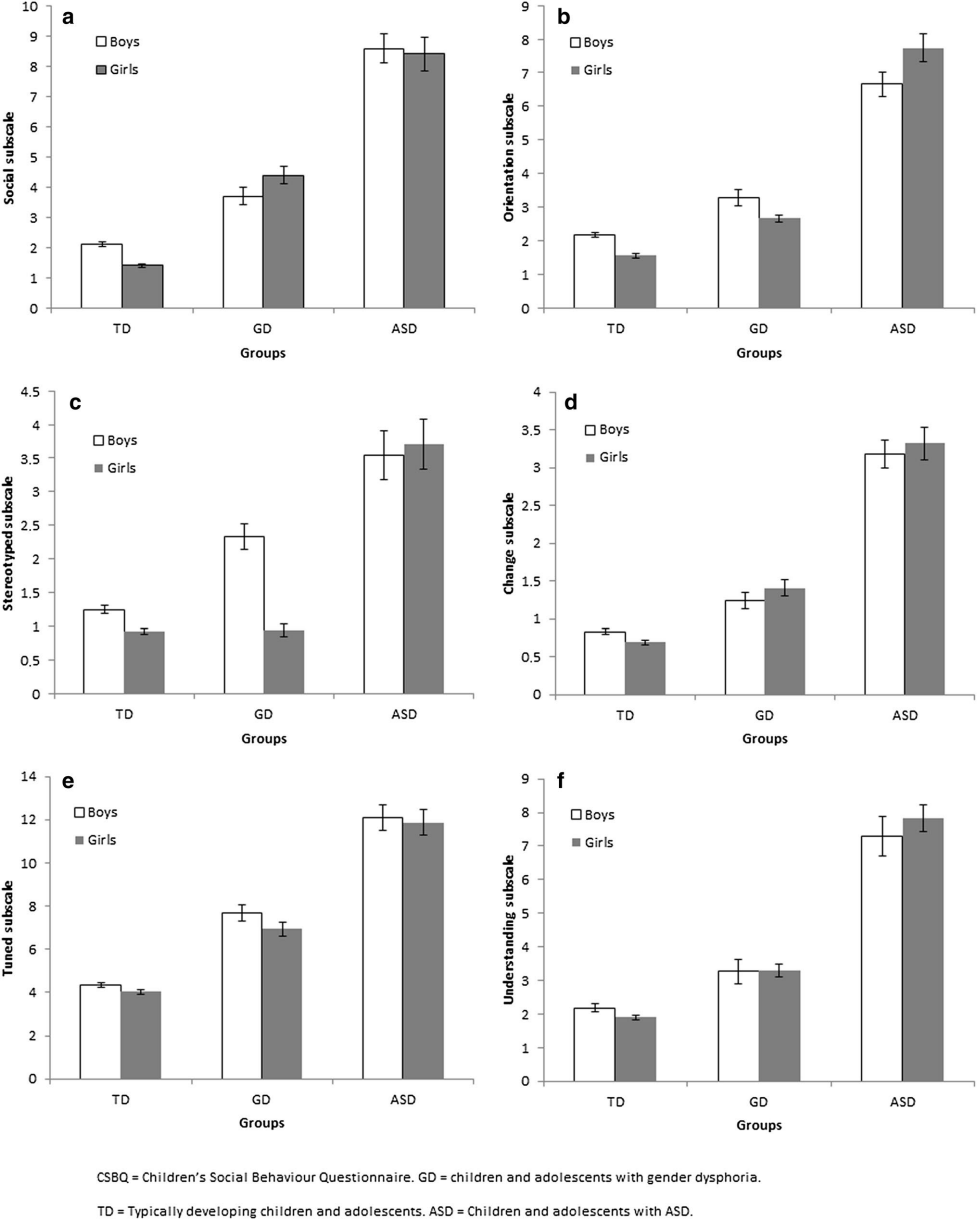
CSBQ mean scores on the six ASD domains in boys and girls with GD compared with TD children and children diagnosed with ASD

On the CSBQ total scale the aforementioned effect of group was confirmed, and there was no main effect of assigned gender at birth nor a group by gender interaction effect. Results of a multivariate GLM analysis on the six CSBQ subscale scores with these predictors also confirmed the aforementioned described group effect. In addition, there was a main effect of assigned gender at birth (*F* = 5.42; *df* = 7; *p* < 0.001) and a group by gender interaction effect (*F* = 8.78; *df *= 14; *p* < 0.001) for the multivariate analysis of the six CSBQ subscale scores. Subsequent univariate analyses indicated that the group by gender interaction effects were present on the subscales *social, orientation, stereotyped* and *change*, as illustrated in Fig. 1, specifically 1a, 1b, 1c and 1d. Group by gender interactions were not found on the subscales *tuned* (*p* = 0.802) and *understanding* (*p* = 0.752) (Fig. 1e, f).

Figure 1 and a within-group post-hoc *t* tests revealed that the interaction arose because in the GD sample, boys had a lower *social* mean score than girls (see Table 2 for mean scores for boys and girls separately for the three groups on all (sub-)scales, test results, and effect sizes), indicating somewhat less reciprocated social behavior and social interest for girls with GD compared to boys with GD (*t* = −1.70; *df* = 488; *p* = 0.089; *d* = −0.15) whereas in the TD group, boys had a higher *social* mean score than girls, indicating less reciprocated behavior and less social interest for TD boys (*t* = 6.61; *df* = 2505; *p* < 0.001; *d* = 0.26). By comparison, boys diagnosed with ASD scored almost similar as girls with ASD (*t* = 0.23; *df* = 194; *p* = 0.811; *d* = 0.03). Thus, on the *social* scale, the higher scores of girls with GD compared to boys with GD were atypical compared to normative gender differences in the TD population.

**Table 2:** Mean male and female CSBQ scores for children and adolescents with gender dysphoria and comparison groups

	Typically developing					Gender dysphoric					Autism spectrum disorder				
Scales^b^	Boys		Girls		Effect sizes Cohen’s *d* ^a^	Boys		Girls		Effect sizes Cohen’s *d* ^a^	Boys		Girls		Effect sizes Cohen’s *d* ^a^
	Mean	SD	Mean	SD		Mean	SD	Mean	SD		Mean	SD	Mean	SD	
Tuned	4.35	4.28	4.04	3.93	0.08	7.68	5.59	6.93	5.40	0.14	12.08	5.95	11.88	5.90	0.03
Social	2.10	2.96	1.40	2.30	0.26	3.69	4.45	4.38	4.52	− 0.15	8.57	4.78	8.40	5.40	0.03
Orientation	2.17	2.63	1.56	2.26	0.25	3.28	3.61	2.66	3.19	0.18	6.66	3.65	7.74	4.15	− 0.28
Understanding	2.18	2.48	1.90	2.18	0.12	3.27	3.20	3.30	3.09	− 0.01	7.29	3.82	7.82	3.85	− 0.14
Stereotyped	1.25	2.02	0.92	1.67	0.18	2.33	2.29	.94	1.57	0.71	3.54	3.60	3.71	3.68	− 0.05
Change	0.83	1.31	0.69	1.12	0.11	1.24	1.76	1.41	1.70	− 0.10	3.18	1.88	3.32	2.15	− 0.07
CSBQ total	12.89	12.43	10.51	10.34	0.21	21.51	16.74	19.62	14.56	0.12	41.32	16.05	42.86	17.46	− 0.09

*CSBQ* Children’s Social Behaviour Questionnaire

^a^Within group effect size differences; Cohen’s *d*: 0.80 or higher is a large effect size, 0.50–0.79 a medium effect size and 0.20–0.49 small. Effect sizes less than 0.20 are negligible (Cohen 1988)

^b^ *Tuned* behavior not optimally changed to the situation, *Social* reduced social interest and contact, *Orientation* orientation problems, in activity, place of time, *Understanding* difficulties in understanding social information, *Stereotyped* stereotyped behavior, *Change* fear or resistance to changes

Table 2 and Fig. 1 show that on the *orientation* subscale in children and adolescents with GD, boys had more orientation problems than girls. A post hoc *t* test revealed that this was a significant difference (*t* = 2.09; *df* = 488; *p* = 0.037; *d* = 0.18). In TD children and adolescents, boys had also more orientation problems than girls (*t* = 6.31; *df* = 2505; *p* < 0.001; *d* = 0.25). In contrast, in children and adolescents diagnosed with ASD, girls had more orientation problems than boys (*t* = −1.93; *df* = 194; *p* = 0.054; *d* = 0.28). The gender differences in children and adolescents with GD were thus like those in TD children and adolescents but differed from children and adolescents with ASD.

Figure 1 shows that on the *stereotyped* scale in the GD group, boys showed more stereotyped behaviors and sensory sensitivity than girls and a post-hoc *t* test showed that this was a significant difference (*t* = 6.57; *df* = 488; *p* < 0.001; *d* = 0.71). This also held for the TD group (*t* = 4.34; *df* = 2505; *p* < 0.001; *d* = 0.18), although the gender difference on *stereotyped* was more pronounced in GD than in TD children and adolescents (*d* = .71 vs. *d* = .18). Conversely, in children diagnosed with ASD, girls and boys had equivalent scores on the *stereotyped* subscale (*t* = −0.32; *df *= 194; *p* = 0.746; *d* = −0.05).

Figure 1 illustrates that in children and adolescents with GD, girls and boys had similar scores on *resistance to change* (*t* = −1.09; *df *= 488; *p* = 0.275; *d* = 0.10). This was similar in the ASD group (*t* = −0.49; *df* = 194; *p* = 0.620; *d* = 0.07). In contrast, in TD children and adolescents, boys showed more *resistance to change* than girls (*t* = 2.84; *df* = 2505; *p* = 0.004; *d* = 0.11). Thus, gender differences in children and adolescents with GD in *resistance to change* were not present, comparable to the absence of differences in those diagnosed with ASD, in contrast to boys scoring higher than girls in TD children and adolescents.

In summary, the gender by group interaction effects indicated that, relative to TD children and adolescents, gender differences within the GD group were atypical on the *social* (GD girls higher than GD boys), *stereotyped* (GD boys substantially higher than GD girls), and resistance to *change* (no differences between GD boys and GD girls vs. TD boys higher than TD girls) scales. This variable pattern for GD boys and GD girls explains the absence of effects of assigned gender at birth on the total CSBQ score.

It is important to emphasize that these gender by group interaction effects occur on top of the main effects of group. As reported for our GLM analyses, and as illustrated in Fig. 1, it holds for boys and girls with GD alike that, compared to TD children and adolescents, their ASD symptoms are higher, and compared to children and adolescents diagnosed with ASD, their ASD symptoms are substantially lower.

### Boys and Girls with Gender Dysphoria Versus Typically Developing Boys and Girls: Cross Gender Analyses

To compare GD boys with TD girls and TD boys, and GD girls with TD boys and TD girls, as has been done in earlier studies to investigate aspects of the EMB theory (Jones et al. 2012; Pasterski et al. 2014), we additionally provide within and cross-gender effect sizes in Table 3. Confirming the analyses, post-hoc *t* tests revealed that on all scales, GD boys scored higher than TD girls (*p* < 0.001) and TD boys (*p* < 0.001), and GD girls scored higher than TD boys (*p* < 0.001) and TD girls (*p* < 0.001).

**Table 3:** Cross-gender analyses with mean male and female CSBQ scores for gender dysphoric children and adolescents and comparison groups

Scales^b^	Girls with GD		TD girls		Effect sizes Cohen’s *d* ^a^	Boys with GD		TD girls		Effect sizes Cohen’s *d* ^a^	Boys with GD		TD boys		Effect sizes Cohen’s *d* ^a^	Girls with GD		TD boys		Effect sizes Cohen’s *d* ^a^
	Mean	SD	Mean	SD		Mean	SD	Mean	SD		Mean	SD	Mean	SD		Mean	SD	Mean	SD	
Tuned	6.93	5.40	4.04	3.93	0.69	7.68	5.59	4.04	3.93	0.86	7.68	5.59	4.35	4.28	0.74	6.93	5.40	4.35	4.28	0.58
Social	4.38	4.52	1.40	2.30	1.07	3.69	4.45	1.40	2.30	0.83	3.69	4.45	2.10	2.96	0.49	4.38	4.52	2.10	2.96	0.70
Orientation	2.66	3.19	1.56	2.26	0.45	3.28	3.61	1.56	2.26	0.68	3.28	3.61	2.17	2.63	0.39	2.66	3.19	2.17	2.63	0.18
Understanding	3.30	3.09	1.90	2.18	0.60	3.27	3.20	1.90	2.18	0.58	3.27	3.20	2.18	2.48	0.42	3.30	3.09	2.18	2.48	0.43
Stereotyped	.94	1.57	0.92	1.67	0.01	2.33	2.29	0.92	1.67	0.79	2.33	2.29	1.25	2.02	0.52	.94	1.57	1.25	2.02	0.16
Change	1.41	1.70	0.69	1.12	0.58	1.24	1.76	0.69	1.12	0.44	1.24	1.76	0.83	1.31	0.29	1.41	1.70	0.83	1.31	0.42
CSBQ total	19.62	14.56	10.51	10.34	0.82	21.51	16.74	10.51	10.34	0.95	21.51	16.74	12.89	12.43	0.65	19.62	14.56	12.89	12.43	0.53

*GD* Children and adolescents with gender dysphoria, *TD* Typically developing children and adolescents, *ASD* Children and adolescents with ASD, *CSBQ* Children’s Social Behaviour Questionnaire

^a^Across group effect size differences; Cohen’s *d*: 0.80 or higher is a large effect size, 0.50–0.79 a medium effect size and 0.20–0.49 small. Effect sizes less than 0.20 are negligible (Cohen 1988)

^b^ *Tuned* behavior not optimally changed to the situation, *Social* reduced social interest and contact, *Orientation* orientation problems in activity, place of time, *Understanding* difficulties in understanding social information, *Stereotyped* stereotyped behavior, *Change* fear or resistance to changes

### Applying a Dichotomous Cut-off for Suggestive ASD

Finally, as in previous studies (Joneset al. 2012; Pasterski et al. 2014), we applied a dichotomous cut-off in order to generate a tentative estimate of ASD in children and adolescents with GD relative to an estimate in the normative population sample. 14.5% of children and adolescents with GD had a threshold score of 38 or higher, potentially suggestive of an ASD diagnosis, compared to 3.5% in our normative sample. No differences were found between the number of GD boys and GD girls above this threshold (χ^2^ = 55.89; *p* = 0.808).

## Discussion

As hypothesized, children and adolescents with GD had, on average, more autistic symptoms compared to TD children and adolescents but less autistic symptoms compared to children and adolescents with ASD. Although the CSBQ is a questionnaire for screening and not a diagnostic instrument, applying the recommended cut-off indicating that further diagnostic research is warranted for a possible clinical diagnosis for ASD (Luteijn et al. 2002), the prevalence was 14.5%, which is approximately four times higher than the 3.5% in the normative sample and also higher than the current prevalence estimate of 1% in the general population (Lai et al. 2014). Thus, as in other studies, an over-representation of symptoms of ASD in children and adolescents with GD was confirmed (Jones et al. 2012; Pasterski et al. 2014; Skagerberg et al. 2015).

However, contrary to our second hypothesis, children and adolescents with GD not only had more stereotyped behavior and resistance to change but also more difficulties in social interest and reciprocity, tuning to social situations, orientation problems and the understanding of social language compared to TD children and adolescents. This is partly in line with the study of VanderLaan et al. (2015b), which suggested that specifically intense obsessional interests are one of the hypothesized mechanisms underlying the possible GD-ASD co-occurrence. Our results point to several subdomains of the autistic spectrum that might be involved in this possible association, including social and communication difficulties as suggested earlier by Strang et al. (2014) and VanderLaan et al. (2015a). Indeed, in clinical practice, we know that adolescents with ASD who have always felt ‘strange’ or ‘different’ compared to their peers may attribute this “strangeness” to having feelings of GD (de Vries and Cohen-Kettenis 2012). Yet, their feelings of being different may possibly have a broader background than GD. It must additionally be noted that our finding that rigidity and repetitive and obsessive behaviors do not take precedence over the other ASD domains does not necessarily imply that these behaviors are not the primary contributing factors to the genesis of GD.

The third hypothesis, the EMB theory (Baron-Cohen 2002), which predicts more ASD symptomatology in girls with GD compared to boys with GD, was not supported with respect to the total CSBQ score where we did not find significant differences between boys and girls nor a gender by group interaction. Rather, it was found that both girls and boys with GD scored significantly higher than TD boys and girls. While for girls with GD and ASD, a high level of prenatal androgen exposure could contribute to the co-occurrence, for boys the co-occurrence of GD and ASD remains unexplained by this theory. There is one study in adults with ASD that showed that women have increased masculine behavioral characteristics compared to control women while men with ASD showed increased feminine behavior (Bejerot and Eriksson 2014). The authors hypothesized that a more masculinized development in women and a more feminized development in men might contribute to the co-occurrence of ASD and GD. An MRI study in the brain might support this notion as it showed attenuated normative gender differences in white matter tracts in ASD (Beacher et al. 2011). The same lack of expected white matter gender differences might be present in children with GD, suggesting that the sex dimorphic development of the brains in both conditions is diminished, but this is currently speculative. In conclusion, we found no gender differences in our study on the total CSBQ, both boys and girls with GD scored higher than TD boys and we found mixed interaction effects which were not all consistent with the EMB theory.

Findings of the study have made us consider that the developmental pathway of co-occurring GD and ASD (symptoms) is different in boys and girls. The different interaction effects on different subdomains of ASD for assigned gender at birth might support this idea. Specifically, in our study, within the GD group, only on the *stereotyped* (and *orientation* subscale; but the effect size was negligible), a gender difference such that boys with GD scored substantially higher than girls with GD (gender differences also substantial in comparison to normative gender differences in TD children and adolescents) was found. Possibly, this reflects a different underlying etiologic contributory factor for GD and ASD in boys and girls related to over-responsivity to stimuli. A study in adults with ASD reporting on sensory over-responsivity to stimuli showed differences from TD adults for each sensory domain (proprioception, vision, hearing, smell, taste and touch; Tavassoli et al. 2014). Specific interests of boys with ASD and GD are often of a feminine origin (e.g., glitter and soft clothing) and might be correlated with a need for specific sensory input or processing (Tateno et al. 2008). As sensory processing is suggested as a key feature in ASD (Ben-Sasson et al. 2007) a shared contributory factor for boys with both GD and ASD might be a neurodevelopmental over or under-responsivity to sensory stimuli. Future studies should therefore investigate (problems with) sensory processing in individuals with GD.

Next to (symptoms of ASD) leading to GD, the converse has been suggested as well. Skagerberg et al. (2015) hypothesized for example that in a sample of children and adolescents with GD the increased rate of autistic symptoms might have stemmed from the GD itself by GD causing social difficulties as people with GD can be subject to high levels of bullying and stigma (Holt et al. 2014). However, while this hypothesis could perhaps explain the reported increased social problems, it does not explain the elevations on the remaining subdomains of the ASD spectrum. It should additionally be stressed that the social problems assesed with the CSBQ are not the more general and common social problems that occur in many psychiatric conditions; they are rather specific for ASD (e.g., “doesn’t understand jokes”; “frequently says things that are not relevant to the conversation”; “barely knows the difference between strangers and familiar people, for example, readily goes with strangers”). Additionally, as suggested by VanderLaan et al. (2015a) there might be shared underlying mechanisms involved. For example, high birthweight may be a marker of shared mechanisms underlying both GD and ASD.

The limitations of the current study are fourfold. First, although our focus was specifically on symptom levels and not on an ASD diagnosis, we need to emphasize that the CSBQ (or any other questionnaire) does not provide an ASD diagnosis. The CSBQ is used for charting the heterogeneous problem profile that characterizes ASD but cannot be used for a formal diagnostic classification of ASD which requires extensive interviewing and observation (Hartman et al. 2006). In this light, the 14.5% prevalence rate estimate based on the rough threshold level indicating a possible ASD diagnosis (but not with false positives filtered out as yet) is likely to be too high; see in particular the study which reported an ASD diagnosis of 7.8%, as based on diagnostic interviews (de Vries et al. 2010). We nonetheless applied this cut-off in keeping with prior studies, which also provided a rough estimate (Jones et al. 2012; Pasterski et al. 2014) and with the aim to emphasize the heterogeneity within GD with regard to the presence of ASD symptomatology. Second, although children and adolescents with GD show an increased amount of autistic symptoms compared to TD peers, no conclusion with regard to causal or time-related pathways can be drawn because the study was cross-sectional. The aforementioned discussion should thus be interpreted as an exploration of possible pathways that are currently highly speculative in light of our own findings, as well as the broader literature. Third, the use of a clinical comparison group other than focused on ASD could have improved the study design. For example, it is possible that not only elevated ASD levels but also elevated ADHD levels occur in GD samples (Strang et al. 2014). In addition, as increased symptom levels of ASD are also found in some other clinical populations such as in individuals with depression (Pine et al. 2008), future studies should therefore include other referred control groups. In line with adding another clinical control group, it should be mentioned that even though the CSBQ is a well validated instrument (de Bildt et al. 2009; Hartman et al. 2006, 2015), there is some discussion on whether screening-instruments for ASD in general are specific enough when applied to other clinical populations (Havdahl et al. 2016) and therefore might overrate the ASD prevalence. Another limitation is that the data of the normative sample were collected around 10 years earlier than those from the participants with GD. However, participants with GD who had been collected earlier differed not significantly with respect to their CSBQ scores compared to those who have been referred later. This finding assured that the recent increase in reported prevalence rates of ASD was not present in our sample with GD when using the CSBQ.

Despite these limitations, the presence of the large sample of individuals with GD, the ASD scores of whom could be firmly anchored relative to the score profile of TD children and adolescents as well as of children and adolescents diagnosed with ASD, are important assets of this study. There is now more evidence for an increase in symptoms of ASD in samples with GD that calls for further study into possible involved etiological factors. Second, the present study was able to differentiate between multiple domains of ASD, showing that elevated ASD symptoms in children and adolescents with GD are not restricted to one subdomain, as has been previously suggested (e.g. de Vries et al. 2010; Williams et al. 1996). A third important asset of our study is that the ASD scores of boys and girls in the TD and ASD norm groups could be used to interpret gender differences in GD. For example, on the *social* and *change* subscales, we showed that although both boys and girls with GD had much milder problems than boys and girls with ASD, there was an absence of gender differences in individuals with GD as observed in individuals with ASD, where this gender difference is normative in TD children.

Finally, focusing on relevant future research steps as well as the broader clinical implications, additional research is needed to provide guidelines for clinical management of individuals with GD and (symptoms of) ASD. In most cases, the diagnostic procedure is complex as it is often difficult to a priori distinguish between GD as a separate condition and a pre-occupation applying to ASD (Parkinson 2014). Strang et al. (2016) provided a first clinical guideline for adolescents with GD and ASD based on expert opinions. However, in terms of treatment, it is unknown whether the effective medical gender reassignment treatment for GD in neurotypical individuals (de Vries et al. 2014) is suitable for individuals with co-occurring ASD. As long as such evidence and specific clinical management protocols are not available, diagnosis and treatment of co-occurring GD and ASD will remain highly demanding. It might be helpful, as suggested by van Schalkwyk et al. (2015) to be attentive to the development of gender in children with ASD from an early age as there is evidence that children with ASD develop a gender identity (Abelson 1981) but it is unkown if their gender development might follow a different pattern or timeline. For example, the hypothesis, as suggested by van Schalkwyk et al. (2015), that gender related concerns might represent a potential developmental process in which individuals with ASD are delayed in their gender identity development compared to typically developing individuals, emphasizes the further need for longitudinal research in individuals with ASD and (symptoms of) GD. Clinically, caregivers should provide help exploring the gender narrative of individuals with ASD (Strang et al. 2016).

In conclusion, we demonstrated more autistic symptoms in children and adolescents with GD compared to TD children and adolescents. However, we found less autistic symptoms compared to children and adolescents with ASD, illustrating the heterogeneity among GD in relation to the presence of ASD symptoms. All subdomains of the spectrum of ASD were increased; the possible association between ASD and GD can thus not be attributed to one subdomain of the spectrum, such as rigidity or intense interests. Our study found no significant differences in CSBQ total score between boys and girls with GD, and diverging gender differences on the subdomains of ASD, which are not all consistent with the EMB theory. It is essential that healthcare workers actively look out not only for rigidity and obsessions, but take account of the complete spectrum of ASD symptoms whenever assessing and treating individuals with GD.
